# Effect of Glenohumeral Internal Rotation Deficit on Shoulder in Baseball Pitchers during Fastball Pitching

**DOI:** 10.3390/ijerph17218211

**Published:** 2020-11-06

**Authors:** Hwai-Ting Lin, Yu-Chuan Lin, You-Li Chou, Hung-Chien Wu, Rong-Tyai Wang, Paul Pei-His Chou

**Affiliations:** 1 Department of Sport Medicine, Kaohsiung Medical University, Kaohsiung 807, Taiwan; whiting@kmu.edu.tw; 2 Department of Medical Research, Kaohsiung Medical University Hospital, Kaohsiung 807, Taiwan; 3 Division of Sport Medicine, Department of Orthopedic Surgery, Kaohsiung Medical University Hospital, Kaohsiung 807, Taiwan; 910095@ms.kmuh.org.tw; 4 Institute of Biomedical Engineering, National Cheng-Kung University, Tainan 701, Taiwan; ylchou@mail.ncku.edu.tw; 5 Department of Engineering Science, National Cheng-Kung University, Tainan 701, Taiwan; eric12378945608@yahoo.com.tw (H.-C.W.); rtwang@mail.ncku.edu.tw (R.-T.W.)

**Keywords:** pitching mechanism, shoulder injury, kinesiology, baseball, motion analyses, glenohumeral internal rotation deficit, kinematic, kinetic, passive marker

## Abstract

Previous studies have reported that pitchers with glenohumeral internal rotation deficit (GIRD) may increase the risk of shoulder injury. However, limited information is available regarding the specific effects of GIRD in baseball pitching. The purpose of this study was to investigate whether baseball pitchers with GIRD change their pitching mechanism. Fifteen baseball pitchers with GIRD and 15 pitchers without GIRD were recruited from university or senior high-school teams. A three-dimensional motion analysis system (Eagle System, Motion Analysis Corporation, Santa Rosa, CA, USA) was used to capture the pitching motion while performing fastball pitches. The kinematics and kinetics of the throwing shoulder and trunk were analyzed based on motion captured data. The Mann–Whitney U test was used to test the differences of the analyzed parameters between two groups. At the instant of ball release, the GIRD group showed lower shoulder external rotation and trunk rotation, and larger shoulder horizontal adduction. In addition, the GIRD group exhibited a significantly larger shoulder inferior force in the cocking and acceleration phase, and a significantly larger internal rotation torque in the acceleration phase. The present results suggested that pitchers with GIRD need stretch training to enlarge joint range of motion, and to improve trunk strength and flexibility to alleviate potential problems associated with pitching in GIRD pitchers.

## 1. Introduction

Baseball pitching involves an extremely large range of motion (ROM) and very high angular velocities in the pitching cycle. The pitching motion was divided into six phases [1,2]; namely windup (i.e., from the beginning of motion until a balanced position); stride (i.e., from a balanced position until lead foot contact (FC)); arm cocking (i.e., from lead FC to the instant of maximum shoulder external rotation (MER)); arm acceleration (i.e., from the instant of MER to the instant of ball release (BR)); arm deceleration (i.e., from the instant of BR until the point of maximum shoulder internal rotation); and follow-through (i.e., from the point of maximum shoulder internal rotation until the end of motion). Previous studies have suggested that when pitching fastball, the shoulder external rotation could reach 170 degrees, and suffer shoulder external rotation torque as high as 70 Nm at the instant of maximal external rotation. Furthermore, at the instant of ball release, the humeral internal rotation angular velocity can reach as much as 7250 deg/s and the maximum shoulder compression force was 1090 N [1,3]. As a result, this imposes tremendous loads and forces on the shoulder joint tissues. The repetitive exposure of the shoulder complex to such speeds and forces may cause the soft tissue and bony architecture around the joint to undergo adaptive change. This change can subsequently alter the ROM of the glenohumeral joint [4,5,6,7] with increased external rotation (ER) of about 9.0 degrees and decreased internal rotation (IR) of about 9.0 degrees in the throwing extremity that may lead to retrotorsion [5]. Excessive deficits in dominant shoulder internal rotation are referred to clinically as glenohumeral internal rotation deficit (GIRD).

The disparity between the IR of the dominant and non-dominant shoulder has received extensive attention in recent years [4,5,6]. Some clinicians have suggested that GIRD is a leading cause of certain shoulder injuries. For example, the increased humeral retrotorsion associated with GIRD may lead to a restraint of the humeral head by the posterior capsule at smaller internal rotations [6,8]. Moreover, the point contact of the humerus on the glenoid shifts postero-superiorly; thereby providing more clearance for greater tuberosity and diminishing the glenohumeral contact point in the antero-inferior aspect of the capsule. This leads in turn to an excessive external rotation ROM [9,10], which causes the biceps anchor to be peeled back under tension and prompts injury to the postero-superior structures; particularly the postero-superior aspect of the labrum. This so-called peel-back progression mechanism permits further laxity of the anterior aspect of the capsule. For throwing athletes, the pathologic cycle potentially culminates in torsional failure of the rotator cuff and superior labral anterior-posterior tear (SLAP) lesions of the shoulder joint [4,9].

Previous studies have documented the change in ROM of the dominant shoulder in overhand throwing athletes, and have suggested that this change may increase the risk of injury [9,11,12]. Moreover, clinical evidence suggests that the bony adaptation associated with humeral retrotorsion results in a posterior shifting of the ROM arc of the glenohumeral joint [6]. If the GIRD baseball pitcher changes his pitching motion due to its joint ROM, or if there are compensatory movements of other limb joints, the loading in joint will be increased and the chance of injury will increase. Previous studies showed that GIRD alters the biomechanics of the throwing arm, resulting in arm passive motion change and decreased shoulder strength [7,13]. Recently, research applied wearable sensor to study the relationship between GIRD and ball velocity, arm speed, and medial elbow torque. The results found that GIRD was not a predictor to medial elbow torque or ball velocity [14]. However, the effects of this change on the baseball pitching mechanism are still unclear. So if the coach can train on the changes of the pitching mechanism caused by the GIRD, it will improve the pitching motion and reduce the possibility of injury. Nakamizo et al. investigated the pitching motion in little league pitchers and found that pitchers with GIRD exhibited a significantly larger shoulder external rotation at the end of the cocking phase than those with no GIRD [15]. The leading pathologic process in GIRD is posterior capsular and rotator-cuff tightness, due to the repetitive cocking that occurs with the overhead throwing motion. Therefore, it is unclear whether this finding can be extended to pitchers with more extensive pitching experience. Accordingly, the present study aims to investigate whether GIRD causes a change in the pitching dynamics of senior league and collegiate pitchers with a longer pitching history.

## 2. Materials and Methods 

### 2.1. Experimental Approach to Problem

In accordance with Burkhart et al. [4], GIRD was defined as a loss of 20° or more in the IR of the throwing shoulder as compared with the non-throwing shoulder. In this study, the pitchers were divided into two groups based on this definition, namely a GIRD group and without GIRD group. Fastball is the most commonly used by a pitcher during a baseball game. The internal rotation angular velocity during the acceleration phase of the fastball is also the fastest among all pitch types [16]. Therefore, in this study, we have focused on investigating the effect of GIRD on shoulder during fastball pitching. The kinematics and kinetics data during fastball pitches with maximum effort were analyzed to find out the pitching mechanism change of the pitchers with GIRD.

### 2.2. Subjects

The inclusion criteria were pitchers from top-tier senior high or collegiate baseball teams in Taiwan, and who were free of pain at the time of testing and without upper extremity injury for a period of at least six months previously. The exclusion criteria were pitchers with past shoulder surgery, joint instability or laxity in the lower extremity. The participants and their parents read and signed an informed consent form approved by the Institutional Review Board (IRB) of the Kaohsiung Medical University Chung-Ho Memorial Hospital (KMUHIRB-SV(I)-20150021).

### 2.3. Procedures

After receiving informed consent, we acquired the physical status, pitching career, and passive IR and ER ROMs of the participants in the dominant and non-dominant shoulders at 90° of abduction. The same physical therapist was measured the pitcher’s joint ROM [17].

All pitching motion was captured in the afternoon. Before the measurement, the pitcher still followed the daily routine, e.g., usually do physical training in the early morning, and technical training in the afternoon, and there was no special diet control. We measured the pitching trials using the pitching mound in a real baseball stadium in order to simulate throwing in an actual baseball game. After stretching and warming up by submaximal throwing at a speed of about 70 km/h, each pitcher threw 15 fastball pitches with maximum effort from the pitching mound toward the catcher. To confirm whether the pitcher threw the ball with maximum effort, we measured the ball velocity in each pitch using a Stalker Sport radar gun (Jugs Sports International Distributors, Tualatin, OR, USA). If the ball speed of a pitch reaches 90% of pitcher’s fastest speed of the month, that is regarded the pitcher with maximum effort. 

**Figure 1:**
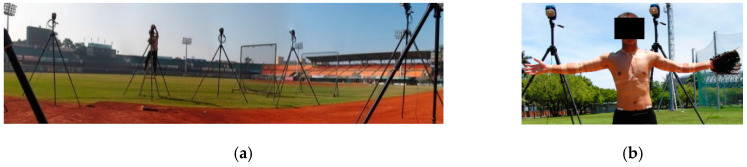
The experimental instrument setting (**a**) cameras setup of the motion capture system in the baseball stadium and (**b**) locations of markers attachments on the subject.

**Figure 2:**
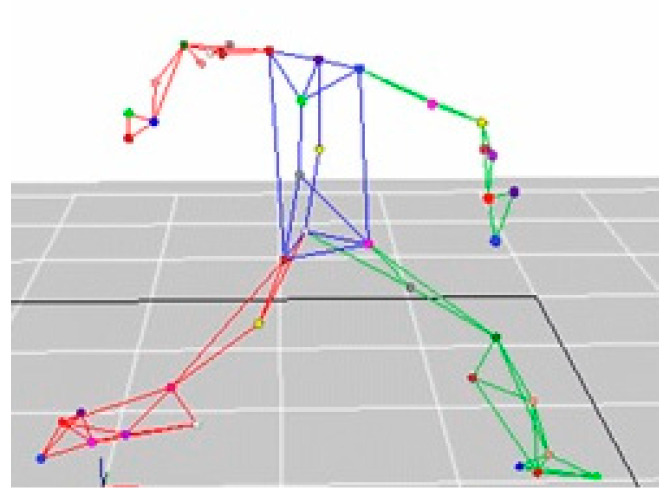
The Cortex software was used for markers’ three-dimensional position tracking.

The distance between the mound and the home plate was set to the standard distance of 18.44 m. A professional umpire standing behind the catcher identified each pitch as a ball or a strike. The pitching motion was captured at a sampling frequency of 300 Hz using a motion analysis system consisting of eight CCD cameras (Eagle System, Motion Analysis Corporation, Santa Rosa, CA, USA) arranged around the pitching mound (Figure 1a). To evaluate the kinematics during the pitching motion, 18 passive markers with reflective stickers (12 mm diameter, Motion Analysis Corporation, Santa Rosa, CA, USA) were attached by trained researchers and using surgical tape (Micropore™, 3M company, Maplewood, MN, USA) to fix on the subjects’ anatomical positions to estimate the joint centers and three-dimensional body-segment locations [18]. The reflective markers were attached on the seventh cervical vertebra, eighth thoracic vertebra, sternum, xiphoid process, marker triad on the right humerus, acromion, lateral epicondyle, medial epicondyle, radius, radial styloid process, ulnar styloid process, third metacarpal bone, and bilateral anterior superior iliac spine and posterior superior iliac spine (Figure 1b). Cortex 2.6 software (Motion Analysis Corporation, Santa Rosa, CA, USA) in the motion analysis system was able to identify the marker’s three-dimensional positions (Figure 2). On completion of the pitching trials, the fastest 5 of the 15 strike pitches thrown by each pitcher were taken for data analysis purposes. Moreover, if ball speed was below 10% of the first five ball speed and pitcher’s Borg’s Rating of Perceived Exertion Scale (RPE, 4–20) [2] was above 11, the pitcher was regarded as tired. 

### 2.4. Theorem

In order to describe the shoulder joint motion and joint loading during the pitching motion, five moving orthogonal coordinate systems (i.e., hand, forearm, upper arm, trunk and pelvis) were defined based on the positions of the reflective markers to specify the relative orientation of each segment [18]. The shoulder joint motion was defined as the movement of the upper arm relative to the trunk. Similarly, the trunk rotation was taken as the movement of the trunk relative to the pelvis. To quantify the shoulder motion, the upper arm was rotated using a z-x’-z” Euler angle (two-axis system) rotation sequence [19]. The first rotation defined the elevation plane (horizontal abduction/adduction) about the z-axis of the humerus. The second rotation defined the humerus elevation/depression about the x’ axis. Finally, the third rotation defined the humerus internal/external rotation about the z’’ axis. The three-axes Eulerian angle rotation system (z-x’-y’’ rotation sequence) was similarly used to quantify the trunk internal/external rotation (z rotation), lateral rotation (x’ rotation) and forward/backward tilt (y’’ rotation) relative to the pelvic frame. All the kinematics and kinetics data were analyzed by the self-coded program in software Matlab 7.0 (The MathWorks, Inc., Natick, MA, USA).

**Figure 3:**
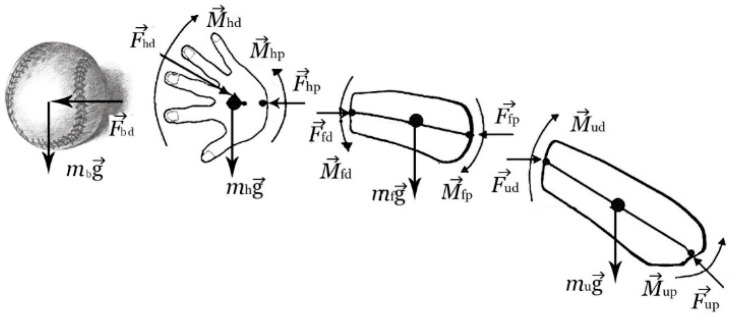
Free body diagram analysis of ball, hand, forearm and upper arm (***F****_p_* is the proximal end joint force; ***F****_d_* is the distal end joint force; *mg* is the gravity force; ***M****_p_* is the proximal end joint moment; ***M****_d_* is the distal end joint moment; u is upper arm; f is forearm; h is hand).

The shoulder joint loading was evaluated using an inverse dynamic Newtonian analysis process. The mass of the baseball was 0.145 kg. More specifically, based on the free-body diagrams of the hand, forearm and upper arm segments shown in Figure 3, the kinematic (i.e., linear and angular displacement, velocity and acceleration) and kinetic (i.e., joint resultant force and torque, and joint work) parameters of the shoulder were quantified. The joint work was defined as joint torque multiple by joint movement. For each participant, the segment masses were calculated simply from the percentage of the total body weight (i.e., upper arm: 2.8%; forearm: 1.6%; hand: 0.6%), and the locations of the centers of mass of the upper arm, forearm and hand were taken respectively as 56.4%, 57.0% and 49.4% of the segment length from the segment distal end [20]. Moreover, the three directions of the segmental moments of inertia were adopted from a previous cadaveric study [21].

In analyzing the captured motion data, the shoulder joint dynamics were investigated from the beginning of the arm cocking phase to the end of the arm acceleration phase. The pitching cycle (PC) was thus defined as the interval between FC (0%PC) and BR (100%PC). 

### 2.5. Statistical Analysis

All data analyses were performed using SPSS statistical software, version 19 (SPSS Inc., Chicago, IL, USA). Shapiro–Wilk test was applied to check the data statistically for normality (*p* > 0.05). However, if the variables were not normally distributed, a non-parametric Mann–Whitney U test was performed for intergroup comparisons. The significance level was set at α = 0.05 (*p* < 0.05). The intraclass correlation coefficient (ICC) was used to check the variability between 5 trials of the analyses data in a subject.

A priori sample size calculation based on anticipated differences in shoulder internal/external rotation ROM as the primary outcome was estimated based on an anticipated large effect (effect size = 0.7) between two groups. The calculation was based on an alpha level of 0.05 and a desired statistical power of 80% using G*Power [22]. The minimum sample size was 15 subjects per group.

## 3. Results

The data acquisition of this study was from 2015/08 to 2016/05. A total of 62 pitchers were interviewed from six top-tier senior high or collegiate baseball teams. Of them, 14 pitchers who did not meet the inclusion criteria were excluded. After shoulder internal/external rotation ROM examination, 15 of the pitchers exhibited GIRD (GIRD group) and the same number of pitchers did not were randomly selected as the normal group. The researchers were blinded with respect to the participant’s medical status, and the subjects did not know which group they belong to.

All of the pitchers were males and right-shoulder dominant. The pitchers in the GIRD group had a mean age of 18.4 ± 2.5 years (range from 16–21 years), a mean height of 178.5 ± 5.5 cm, a mean weight of 77.9 ± 10.8 kg, and a mean playing experiences of 8.6 ± 1.7 years. Meanwhile, those in the non-GIRD group had a mean age of 17.8 ± 2.3 years, a mean height of 176.6 ± 4.7 cm, a mean weight of 73.1 ± 9.2 kg, and a mean playing experiences of 7.8 ± 0.7 years. None of the subjects felt tired during testing. The ICC values between 5 trials of the analyses data in a subject were between 0.736 to 0.885, it showed a high repeatability pitching motion between trials in each testing subject.

Table 1 shows the passive IR ROM of the dominant and non-dominant shoulder in the GIRD and Normal groups. For both groups, the mean passive ROM of IR of the dominant shoulder is significantly lower than that of the non-dominant shoulder. However, the mean deficit of the passive IR ROM in the dominant shoulder is greater in the GIRD group (22.1 degrees) than in the Normal group (8.4 degrees).

No significant difference was found between the mean pitching ball speed in the GIRD group (34.4 ± 2.0 m/s) and the Normal group (33.5 ± 1.5 m/s). However, at the instant of ball release, the shoulder external rotation in the GIRD group was significantly lower than that in the Normal group. In addition, the shoulder horizontal adduction of the GIRD group was significant greater than that of the Normal group. The duration of the arm acceleration phase in the GIRD group was significantly longer than that in the Normal group (Table 2).

The shoulder inferior force of the GIRD group was significantly higher in the GIRD group than in the Normal group in both the cocking phase and the acceleration phase. Moreover, the shoulder internal rotation torque was also significantly greater in the GIRD group than in the Normal group in the acceleration phase (Table 3).

No significant difference was found between the two groups in the rotation work done by the shoulder joint in the cocking phase. However, in the acceleration phase, the internal rotational work and total rotational work of the shoulder joint were significantly higher in the GIRD group than in the Normal group (Table 3).

## 4. Discussion

Since King et al. [23] first reported that the pitching arm of elite pitchers undergoes a greater shoulder external rotation ROM and a lower internal rotation ROM than the non-pitching arm, many investigators have identified differences in the glenohumeral rotational ROM between the dominant and non-dominant shoulders of throwing athletes [5,6]. Our study has also found that the subjects in both groups (GIRD and Normal) undergo a larger shoulder external rotation ROM and lower shoulder internal rotation ROM in the dominant arm than in the non-dominant arm. Interestingly, while the lower ROM of the internal rotation is larger in the GIRD group than in the Normal group, no difference exists between the two groups in the increased external rotation ROM. In general, the present results suggest that pitchers with GIRD experience greater retrotorsion, which leads to a restraint of the humeral head by the posterior capsule [6,24].

The present findings confirm that baseball pitchers with GIRD modify their pitching mechanics. In particular, the results show that GIRD pitchers have a significantly higher shoulder elevation angle and almost reached significant less horizontal abduction angle at the instant of foot contact. Before the instant of MER, the shoulder had changed to the horizontal adduction angle and the shoulder horizontal adduction angle in the GIRD group is greater than that in the Normal group at the instants of BR. This phenomenon is consistent with previous findings regarding the contracture of the posteroinferior capsule during baseball pitching [4,5], and suggests that GIRD pitchers are unable to fully extend the throwing arm at the instant of FC and thus exhibit a greater shoulder horizontal adduction angle at the instants of BR. The present results also show that a difference exists between the two groups in the ROM arc of the shoulder horizontal adduction/abduction angle during pitching. For example, in the GIRD group, the shoulder horizontal angle changes from 23.3 degrees adduction to 5.8 degrees abduction between FC and BR, while in the Normal group, the shoulder horizontal angle changes from 36.1 degrees adduction to 0.6 degrees abduction. The range of shoulder horizontal adduction/abduction motion (i.e., 29.1 degrees in the GIRD group and 36.7 degrees in the Normal group) is significantly different. The lower range of shoulder horizontal adduction/abduction motion in the GIRD group suggests a thickening of the soft shoulder tissue structure, which further affects the pitching mechanism. 

Previous epidemiological studies have indicated that GIRD pitchers exhibit a greater external rotation angle of the dominant shoulder as a result of anterior capsular laxity, posterior capsular tightness and humerus retrotorsion [5,6]. In the present study, the shoulder external rotation in the GIRD group is almost the same as that in the Normal group before the instant of MER. However, the GIRD group shows a lower external rotation angle at the instant of BR. As a result, the acceleration phase has a longer duration in the GIRD group than in the Normal group. Impulse theory in physics tells that a force applied over a longer time could produce a larger change in linear momentum, and therefore it suggests that a longer acceleration phase should result in a higher ball speed at the moment of release. However, no significant difference was found in the ball velocity between the two groups in the present study. This may indicate that the Normal group applies a shoulder horizontal adduction movement to accelerate the pitching arm instead of the need for longer acceleration. Notably, a longer acceleration phase tends to increase the shoulder loading; particularly following repeated pitches during a game. Thus, the risk of shoulder injury also increases due to a change in the pitching mechanism.

**Figure 4:**
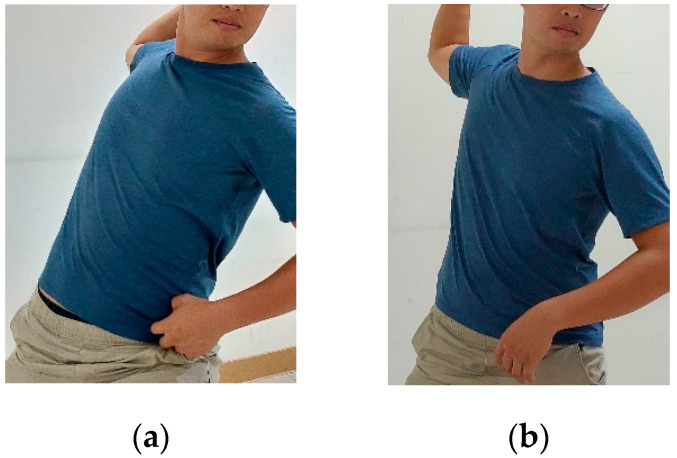
The contract of the torso rotation between two groups. (**a**) Normal group with larger torso rotation (**b**) GIRD group with less torso rotation.

Optimizing the orientation and rotational velocity of the pelvis and torso during baseball pitching enhances momentum generation, and thus allows the pitcher to transfer a greater amount of energy through the kinetic chain from the trunk to the throwing arm [25]. As a result, the ball velocity increases. The present results show that the GIRD group performs significantly lower trunk internal rotation than the Normal group from the instant of FC to that of MER and BR. This implies that the Normal group utilizes upper torso rotation to generate and transfer energy to the throwing arm, and thus reduces the burden on the shoulder and elbow joint during pitching. By contrast, the GIRD group depends more heavily on the upper arm (i.e., the shoulder and elbow joint) during throwing motion due to a lack of momentum transfer through the kinetic chain (Figure 4). This heavier loading potentially leads to cumulative micro-trauma of the shoulder joint following repeated pitching motions, and hence increases the risk of shoulder injury [26]. 

The present findings show that the shoulder inferior force in the GIRD group is significantly greater than that in the Normal group in both the arm cocking phase and the arm acceleration phase. Overall, the results show that the shoulder of the GIRD group bears a high load, which results in a tightness of the posterior capsule during the arm cocking phase. The shoulder internal rotation torque of the GIRD group is also significantly greater than that of the Normal group in the acceleration. These are increasing the possibility of internal impingement and instability of the shoulder [27]. Snyder et al. [28] reported that superior labral injury can be attributed to the combined effects of a compression force on the superior joint surface and a proximal subluxation force on the humeral head. The resulting stresses may cause a traumatic disruption of the labrum and a possible compression fracture of the superior humeral head. The present findings are consistent with epidemiological study and suggest that GIRD pitchers may be prone to labral injury and rotator cuff tear [28].

Achieving fastball speeds during throwing requires a combination of elastic energy storage at the shoulder and efficient kinetic energy transfer from the proximal body segments to the distal segments. The findings presented in this study for the work done by the shoulder joint are consistent with those reported previously for the upper extremity kinetic chain mechanism [29]. More specifically, the present results show that the shoulder joint in the GIRD group performs greater rotational work than that in the Normal group during the acceleration phase. Notably, however, the greater work performed by the shoulder in the GIRD group does not contribute to a significantly faster throwing speed, but simply increases the load acting on the shoulder. 

In this study, the pitchers pitched in a real pitching mount and in a real baseball stadium that could simulate throwing in an actual baseball game. All kinematics and kinetics data analyses about the shoulder could help us better understand the dynamic pitching mechanism. However, there were some limitations of this study. Data were analyzed from the marker positions captured from the motion system, and the relative movement between marker on the skin and bony landmark was not considered in this study. Due to the relatively small sample size of the GIRD and Normal groups, care should be taken in generalizing the present findings to a wider population. All of these pitchers were from top-tier senior high or collegiate baseball teams in Taiwan, and have a certain level of pitching performance. Classification of the two groups has been performed solely on the basis of the deficits of the internal rotation angle of the dominant and non-dominant shoulders, respectively; the pitching experience of the participants has not been considered when dividing the groups. Moreover, the lack of pathological examination to identify whether the pitchers have an injury or the location of the injury is a limitation of this study. Finally, the present analyses have considered only fast ball pitches. In other words, pitches such as slider and change-up have been ignored. 

This study has shown that baseball pitchers with GIRD exhibit a modified pitching mechanism. In particular, GIRD pitchers show a greater horizontal adduction angle and reduced external rotation angle than non-GIRD pitchers and, consequently, have a different ball release position. In addition, GIRD pitchers have a smaller torso rotation angle, a larger shoulder inferior force, a greater shoulder internal rotation torque, and increased internal rotation work of the shoulder joint. In general, GIRD pitchers fail to properly transfer power from the trunk to the throwing arm, and thus rely more heavily on the shoulder joint during pitching. Consequently, the risk of shoulder injury is increased. The present results therefore confirm the assertion of previous clinical studies that pathologic GIRD is a major cause of shoulder problems in throwing athletes [9,11,12]. 

## 5. Conclusions

This study found that GIRD pitchers fail to properly transfer power from the trunk to the throwing arm, and thus rely more heavily on the shoulder joint during pitching. Consequently, the risk of shoulder injury is increased. Larger loading on the shoulder during the pitching motion in GIRD pitchers may increase the risk of injury. The findings of this study also provide useful information for pitchers and coaches in adjusting the pitching motion so as to alleviate potential shoulder problems of the throwing arm. In addition, the results suggest that improving the trunk strength and flexibility is desirable in order to improve the kinetic energy transfer efficiency from the proximal body segments to the distal segments during the late cocking to acceleration phases of the pitching motion. 

## Figures and Tables

**Table 1:** Passive range of motion of dominant and non-dominant shoulders.

ROM	GIRD			Normal		
	Dominant	Non-Dominant	*p*-Value	Dominant	Non-Dominant	*p*-Value
Shoulder internal rotation (°)	41.9 ± 12.4	64.0 ± 16.9	<0.001	44.0 ± 15.6	52.4 ± 15.4	<0.001
Shoulder external rotation (°)	138.2 ± 9.6	114.2 ± 11.0	<0.001	136.7 ± 6.3	116.5 ± 9.5	<0.001
**Internal rotation deficit of dominant (°)**	**22.1 ± 7.2**			**8.4 ± 4.2**		

Data reported as Mean ± standard deviation. GIRD, glenohumeral internal rotation deficit; Bold values are statistically significance differences between groups, *p* *<* 0.05.

**Table 2:** Comparison of kinematic parameters in GIRD and Normal groups.

Kinematics Parameters	GIRD	Normal	*p*-Value	Note
**Instant of lead foot contact**				
Shoulder horizontal abduction (°)	23.3 ± 16.3	36.1 ± 12.1	0.068	
** Shoulder elevation (°)**	**88.3 ± 12.0**	**76.3 ± 11.0**	**0.024**	**G > N**
Shoulder external rotation (°)	41.7 ± 18.0	35.1 ± 39.4	0.738	
**Instant of maximum shoulder external rotation**				
Shoulder horizontal adduction (°)	7.3 ± 8.0	2.8 ± 6.7	0.138	
Shoulder elevation (°)	91.2 ± 8.9	86.6 ± 7.5	0.229	
Shoulder external rotation (°)	159.2 ± 8.7	159.0 ± 11.6	0.960	
Trunk tilt lateral (°)	29.6 ± 32.9	32.9 ± 8.9	0.424	
** Trunk internal rotation (°)**	**2.3 ± 6.0**	**11.5 ± 7.1**	**<0.001**	**N > G**
Duration time_ FC-MER (ms)	120.1 ± 25.9	123.9 ± 26.7	0.303	
**Arm acceleration phase**				
Max shoulder internal rotation angular velocity (°/sec)	6065.3 ± 1380.4	6588.9 ± 1126.8	0.349	
Max shoulder horizontal adduction angular velocity (°/sec)	709.1 ± 352.3	749.4 ± 348.9	0.790	
** Duration time (ms)**	**30.4 ± 4.9**	**22.0 ± 5.7**	**<0.001**	**G > N**
**Instant of ball release**				
** Shoulder horizontal adduction (°)**	**5.8 ± 5.0**	**0.6 ± 6.1**	**0.048**	**G > N**
Shoulder elevation (°)	84.0 ± 6.3	81.3 ± 8.3	0.426	
** Shoulder external rotation (°)**	**104.4 ± 16.2**	**119.4 ± 13.2**	**0.026**	**N > G**
** Trunk internal rotation (°)**	**12.8 ± 6.1**	**19.0 ± 6.1**	**0.034**	**N > G**
Duration (FC-BR) (ms)	150.5 ± 28.4	146.0 ± 27.7	0.361	

Values are expressed as mean ± standard deviation. G, glenohumeral internal rotation deficit group; N, normal group; FC-MER, foot contact to maximum external rotation; FC-BR, foot contact to ball release; Bold values are statistically significance differences between groups, *p* *<* 0.05.

**Table 3:** Comparison of kinetic parameters in GIRD and Normal groups.

Kinetics Parameters	GIRD	Normal	*p*-Value	Note
**Arm cocking phase**				
Shoulder posterior force (N)	147.6 ± 36.6	165.8 ± 29.2	0.142	
** Shoulder inferior force (N)**	**297.5 ± 54.6**	**245.7 ± 50.2**	**0.048**	**G > N**
Shoulder proximal force (N)	454.2 ± 116.7	520.9 ± 82.8	0.184	
** Shoulder adduction torque (Nm)**	**52.7 ± 9.6**	**43.4 ± 8.6**	**0.037**	**G > N**
Shoulder internal rotation torque (Nm)	42.9 ± 8.3	37.5 ± 6.1	0.065	
Shoulder internal rotation work (Joule)	−61.7 ± 16.9	−54.2 ± 10.6	0.272	
Shoulder resultant work (Joule)	−82.7 ± 24.1	−66.2 ± 22.6	0.108	
Shoulder internal rotation work (Joule)	−61.7 ± 16.9	−54.2 ± 10.6	0.272	
**Arm acceleration phase**				
Shoulder posterior force (N)	227.1 ± 47.9	238.3 ± 59.0	0.630	
** Shoulder inferior force (N)**	**84.2 ± 55.8**	**12.2 ± 67.2**	**0.016**	**G > N**
Shoulder proximal force (N)	627.7 ± 104.8	635.1 ± 85.9	0.867	
** Shoulder elevation torque (Nm)**	**6.4 ± 14.5**	**23.2 ± 20.2**	**0.032**	**N > G**
** Shoulder internal rotation torque (Nm)**	**32.0 ± 9.3**	**19.2 ± 8.7**	**0.002**	**G > N**
Shoulder elevation work (Joule)	−1.3 ± 2.7	−0.3 ± 1.2	0.389	
Shoulder horizontal abduction work (Joule)	0.3 ± 1.3	0.2 ± 0.8	0.870	
** Shoulder internal rotation work (Joule)**	**8.4 ± 5.8**	**1.5 ± 4.7**	**0.006**	**G > N**
** Shoulder resultant work (Joule)**	**7.5 ± 5.0**	**1.4 ± 5.2**	**0.006**	**G > N**

Values are expressed as mean ± standard deviation. G, glenohumeral internal rotation deficit group; N, normal group; Bold values are statistically significance differences between groups, *p* *<* 0.05.

## References

[1] Fleisig G.S., Barrentine S.W., Escamilla R.F., Andrews J.R. (1996). Biomechanics of overhand throwing with implications for injuries. Sports Med. Auckl. N. Z..

[2] Chou P.P.H., Huang Y.P., Gu Y.H., Liu C., Chen S.K., Hsu K.C., Wang R.T., Huang M.J., Lin H.T. (2015). Change in pitching biomechanics in the late-inning in Taiwanese high school baseball pitchers. J. Strength Cond. Res. Natl. Strength Cond. Assoc..

[3] Fleisig G.S., Andrews J.R., Dillman C.J., Escamilla R.F. (1995). Kinetics of baseball pitching with implications about injury mechanisms. Am. J. Sports Med..

[4] Burkhart S.S., Morgan C.D., Kibler W.B. (2003). The disabled throwing shoulder: Spectrum of pathology Part I: Pathoanatomy and biomechanics. Arthrosc. J. Arthrosc. Relat. Surg..

[5] Crockett H.C., Gross L.B., Wilk K.E., Schwartz M.L., Reed J., O’Mara J., Reilly M.T., Dugas J.R., Meister K., Lyman S. (2002). Osseous adaptation and range of motion at the glenohumeral joint in professional baseball pitchers. Am. J. Sports Med..

[6] Reagan K.M., Meister K., Horodyski M.B., Werner D.W., Carruthers C., Wilk K. (2002). Humeral retroversion and its relationship to glenohumeral rotation in the shoulder of college baseball players. Am. J. Sports Med..

[7] Amin N.H., Ryan J., Fening S.D., Soloff L., Schickendantz M.S., Jones M. (2015). The Relationship Between Glenohumeral Internal Rotational Deficits, Total Range of Motion, and Shoulder Strength in Professional Baseball Pitchers. J. Am. Acad. Orthop. Surg..

[8] Jobe C.M., Iannotti J.P. (1995). Limits imposed on glenohumeral motion by joint geometry. J. Shoulder Elb. Surg..

[9] Burkhart S.S., Morgan C.D., Kibler W.B. (2003). The disabled throwing shoulder: Spectrum of pathology. Part II: Evaluation and treatment of SLAP lesions in throwers. Arthrosc. J. Arthrosc. Relat. Surg..

[10] Tyler T.F., Nicholas S.J., Roy T., Gleim G.W. (2000). Quantification of posterior capsule tightness and motion loss in patients with shoulder impingement. Am. J. Sports Med..

[11] Wilk K.E., Macrina L.C., Fleisig G.S., Aune K.T., Porterfield R.A., Harker P., Evans T.J., Andrews J.R. (2015). Deficits in Glenohumeral Passive Range of Motion Increase Risk of Shoulder Injury in Professional Baseball Pitchers: A Prospective Study. Am. J. Sports Med..

[12] Garrison J.C., Cole M.A., Conway J.E., Macko M.J., Thigpen C., Shanley E. (2012). Shoulder range of motion deficits in baseball players with an ulnar collateral ligament tear. Am. J. Sports Med..

[13] Gates J.J., Gupta A., McGarry M.H., Tibone J.E., Lee T.Q. (2012). The effect of glenohumeral internal rotation deficit due to posterior capsular contracture on passive glenohumeral joint motion. Am. J. Sports Med..

[14] Smith D.G., Swantek A.J., Gulledge C.M., Lizzio V.A., Bermudez A., Schulz B.M., Makhni E.C. (2019). Relationship Between Glenohumeral Internal Rotation Deficit and Medial Elbow Torque in High School Baseball Pitchers. Am. J. Sports Med..

[15] Nakamizo H., Nakamura Y., Nobuhara K., Yamamoto T. (2008). Loss of glenohumeral internal rotation in little league pitchers: A biomechanical study. J. Shoulder Elb. Surg..

[16] Fleisig G.S., Kingsley D.S., Loftice J.W., Dinnen K.P., Ranganathan R., Dun S., Escamilla R.F., Andrews J.R. (2006). Kinetic comparison among the fastball, curveball, change-up, and slider in collegiate baseball pitchers. Am. J. Sports Med..

[17] Reinold M.M., Wilk K.E., Macrina L.C., Sheheane C., Dun S., Fleisig G.S., Crenshaw K., Andrews J.R. (2008). Changes in shoulder and elbow passive range of motion after pitching in professional baseball players. Am. J. Sports Med..

[18] Wu G., van der Helm F.C., Veeger H.E., Makhsous M., Van Roy P., Anglin C., Nagels J., Karduna A.R., McQuade K., Wang X. (2005). ISB recommendation on definitions of joint coordinate systems of various joints for the reporting of human joint motion--Part II: Shoulder, elbow, wrist and hand. J. Biomech..

[19] An K.N., Browne A.O., Korinek S., Tanaka S., Morrey B.F. (1991). Three-dimensional kinematics of glenohumeral elevation. J. Orthop. Res..

[20] Winter D.A. (2005). Biomechanics and Motor Control of Human Movement.

[21] de Leva P. (1996). Adjustments to Zatsiorsky-Seluyanov’s segment inertia parameters. J. Biomech..

[22] Faul F., Erdfelder E., Lang A.G., Buchner A. (2007). G*Power 3: A flexible statistical power analysis program for the social, behavioral, and biomedical sciences. Behav. Res. Methods.

[23] King J.W., Brelsford H.J., Tullos H.S. (1969). Analysis of the pitching arm of the professional baseball pitcher. Clin. Orthop. Relat. Res..

[24] Noonan T.J., Shanley E., Bailey L.B., Wyland D.J., Kissenberth M.J., Hawkins R.J., Thigpen C.A. (2015). Professional Pitchers With Glenohumeral Internal Rotation Deficit (GIRD) Display Greater Humeral Retrotorsion Than Pitchers Without GIRD. Am. J. Sports Med..

[25] Stodden D.F., Fleisig G.S., McLean S.P., Lyman S.L., Andrews J.R. (2001). Relationship of pelvis and upper torso kinematics to pitched baseball velocity. J. Appl. Biomech..

[26] Ryu R.K., Dunbar W.H., Kuhn J.E., McFarland E.G., Chronopoulos E., Kim T.K. (2002). Comprehensive evaluation and treatment of the shoulder in the throwing athlete. Arthrosc. J. Arthrosc. Relat. Surg..

[27] Myers J.B., Laudner K.G., Pasquale M.R., Bradley J.P., Lephart S.M. (2006). Glenohumeral range of motion deficits and posterior shoulder tightness in throwers with pathologic internal impingement. Am. J. Sports Med..

[28] Snyder S.J., Karzel R.P., Del Pizzo W., Ferkel R.D., Friedman M.J. (1990). SLAP lesions of the shoulder. Arthrosc. J. Arthrosc. Relat. Surg..

[29] Roach N.T., Venkadesan M., Rainbow M.J., Lieberman D.E. (2013). Elastic energy storage in the shoulder and the evolution of high-speed throwing in Homo. Nature.

